# MR lymphangiography of lymphatic abnormalities in children and adults with Noonan syndrome

**DOI:** 10.1038/s41598-022-13806-w

**Published:** 2022-07-01

**Authors:** C. C. Pieper, J. Wagenpfeil, A. Henkel, S. Geiger, T. Köster, K. Hoss, J. A. Luetkens, C. Hart, U. I. Attenberger, A. Müller

**Affiliations:** 1 Department of Diagnostic and Interventional Radiology, University of Bonn, Venusberg-Campus 1, 53127 Bonn, Germany; 2 Department of Pediatric Cardiology, Children’s Hospital, University of Bonn, Bonn, Germany; 3 Department of Neonatology and Pediatric Intensive Care, Children’s Hospital, University of Bonn, Bonn, Germany

**Keywords:** Genetics, Medical research, Respiratory tract diseases

## Abstract

Noonan syndrome is associated with complex lymphatic abnormalities. We report dynamic-contrast enhanced MR lymphangiography (DCMRL) findings in children and adults with Noonan syndrome to further elucidate this complex disease spectrum. A retrospective evaluation of patients with confirmed Noonan syndrome and clinical signs of lymphatic dysfunction undergoing DCMRL between 01/2019 and 04/2021 was performed. MRL included T2-weighted imaging (T2w) and DCMRL. Clinical history/presentation and genetic variants were recorded. T2w-imaging was evaluated for central lymphatic abnormalities and edema distribution. DCMRL was evaluated regarding the presence of cisterna chyli/thoracic duct, lymphatic leakages, pathological lymphatic reflux and abnormal lymphatic perfusion. The time from start of contrast-injection to initial enhancement of the thoracic duct venous junction was measured to calculate the speed of contrast propagation. Eleven patients with Noonan syndrome with lymphatic abnormalities (5 female, 6 male; 7 infants, 4 adults; mean age 10.8 ± 16.4 years) were identified (*PTPN11* n = 5/11 [45.5%], *RIT1* n = 5/11 [45.5%], KRAS n = 1/11 [9%]). Patients had a chylothorax (n = 10/11 [91%]) and/or pulmonary lymphangiectasia [dilated pulmonary lymph vessels] (n = 9/11 [82%]). Mediastinal/pulmonary edema was depicted in 9/11 (82%) patients. The thoracic duct (TD) was (partially) absent in 10/11 (91%) cases. DCMRL showed lymphatic reflux into intercostal (n = 11/11 [100%]), mediastinal (n = 9/11 [82%]), peribronchial (n = 8/11 [73%]), peripheral (n = 5/11 [45.5%]) and genital lymphatics (n = 4/11 [36%]). Abnormal pulmonary/pleural lymphatic perfusion was seen in 8/11 patients (73%). At infancy peripheral/genital edema was more prevalent in patients with *RIT1* than *PTPN11* (n = 3/5 vs. n = 0/5). Compared to patients with *PTPN11* who had fast lymphatic enhancement in 4/5 patients, enhancement took markedly longer in 4/5 patients with *RIT1*-mutations. Thoracic duct dysplasia, intercostal reflux and pulmonary/pleural lymphatic perfusion are characteristic findings in patients with Noonan syndrome presenting with chylothorax and/or pulmonary lymphangiectasia. Central lymphatic flow abnormalities show possible phenotypical differences between *PTPN11* and *RIT1*-mutations.

## Introduction

Noonan syndrome is an autosomal dominant, genetically heterogeneous disorder belonging to the so called RASopathies^1,2^. A number of causative germ-line mutations in several genes has been identified with the major genes being *PTPN11* (approx. 50% of cases), *RIT1*, *SOS1* and *RAF1*^2–8^. Characteristic clinical features include short stature/growth retardation, facial dysmorphisms, pterygium colli, skeletal abnormalities and congenital heart disease^1^.

Noonan syndrome has also been associated with lymphatic abnormalities in approximately 15–20% of cases, including lymphedema, pulmonary lymphangiectasia (i.e. dilated pulmonary lymph vessels) and chylothorax^2,9–17^. Lymphatic anomalies can be present in the neonatal period or develop later in life (e.g. late-onset lymphedema)^2,15^.

Although lymphatic abnormalities in Noonan syndrome have been recognized on lymphatic imaging for decades^13,18^, data are limited to case reports and small case series^13,14,19–25^. With the recent introduction of dynamic contrast-enhanced MR lymphangiography (DCMRL) new imaging options have become available offering less invasive evaluation of lymphatic abnormalities^26–29^. Recently initial experiences with DCMRL in a pediatric population with Noonan syndrome showed intercostal lymphatic reflux, abnormal pulmonary lymphatic perfusion and thoracic duct (TD) abnormalities in a number of patients^17^.

In the following, we want to report our MR-imaging experiences in children and adults with Noonan syndrome—with different gene variants—suffering from lymphatic abnormalities to further elucidate this complex disease spectrum.

## Materials and methods

### Inclusion criteria

Patients were included into the study when they had.

- Confirmed Noonan syndrome,
- A history of lymphatic abnormalities (lymphedema, chylothorax, chylous ascites, pulmonary or intestinal lymphangiectasia, lymphorrhea),
- A clinical indication for DCMRL and
- Had undergone examination at our institution between 01/2019 and 04/2021.

DCMRL was performed as part of our standard clinical work-up in patients with suspected lymphatic pathologies. Patients / parents were informed about the procedure in detail especially off-label use of the MR contrast-agent for lymphangiography and provided written informed consent for the examination.

### Imaging technique

MR-examinations were performed on a 1.5-T system (Ingenia; Philips Healthcare, Best, The Netherlands) as described in details elsewhere^28^. In short, the patient was placed on a detachable MR table in supine position. First, non-contrast heavily T2-weighted (T2w) 3D-imaging was acquired using a free-breathing 3D-high-spatial-resolution, isotropic turbo spin-echo-sequence (TR 3000 ms, TE 600 ms, flip angle 90°, field of view 400 mm, acquired voxel size 1.2 × 1.2 × 1.2 mm, reconstructed voxel size 0.6 × 0.6 × 0.6 mm, acquisition time 4:45 min). Next, the patients were transferred out of the scanner room and inguinal lymph nodes were identified sonographically using a linear 18 MHz probe (LOGIQ Vivid E90, GE Healthcare). Under sterile conditions, the tip of a 25-gauge spinal needle (BD Medical, USA) was positioned in the lymph node. All ultrasound-guided lymph node punctures were performed bilaterally by the same interventional radiologist (C.C.P. with 10 years of interventional experience). With satisfactory needle position patients were transferred back into the MR-scanner and DCMRL was performed with continuous slow application (adults: 1 ml per minute, infants: 0.5 ml per minute) of diluted 1.0 mmol/mL gadobutrol (Gadovist, Bayer Healthcare, Germany; diluted 1:4 with physiological saline; 0.05 ml/kg body-weight) by hand. To ensure comparable contrast application, injection was performed by one of the same two radiologists (J.W. or S.G.) with a clock available for steady application at the desired flow rate. During contrast-injection a coronal T1-weighted multi-echo gradient-echo-sequence was repetitively acquired at time intervals of 45 s (TR 5.2 ms, TE 1.8 ms and 4 ms, flip angle 20°, field of view: 430 mm, matrix: 480 × 480 mm, acquisition time 40 s). Young children were examined under general anesthesia. Peri-interventional complications were recorded if present.

### Data analysis and definitions

Medical records were retrospectively reviewed to gather relevant medical history including the gene variant, associated congenital heart disease and the clinical history of lymphatic abnormalities during infancy (< 1 year), childhood (> 1 year, < 18 years) and adulthood (if available). Measured triglyceride levels above 110 mg/dl on laboratory examination (while the patients was on normal enteral nutrition) were used as a surrogate parameter to define effusions as chylous^30^.

MR lymphangiograms were retrospectively reviewed by two radiologists in consensus (C.C.P and J.W. with 10 and 5 years of experience, respectively) blinded to patients’ clinical data.

T2w-imaging was evaluated for:

- Presence of cisterna chyli/TD
- Ascites, pleural/pericardial effusions,
- Edema of the periphery (arms/legs), genital, abdominal wall, mesentery, liver/periportal space, thoracic wall, mediastinum/hilum, lungs, neck and axilla.

Dynamic-contrast-enhanced MR lymphangiograms (DCMRL) were evaluated regarding:

- Presence of cisterna chyli/TD
- Lymphatic leakage
- Pathological lymphatic reflux into lymphatics of the periphery, genital, abdominal wall, mesentery, liver, mediastinum/mediastinal fat, bronchial wall/lung parenchyma, intercostal spaces, internal mammary, superficial thorax, neck and axilla,
- Abnormal lymphatic perfusion of peritoneum, pleura and/or lung parenchyma.

For imaging analysis, the TD was divided into a lower, middle and upper thoracic part, a cervical part as well as the TD-venous junction. Lymphatic reflux was defined as reversal of lymph flow away from central lymphatics/the TD and the TD-venous junction.

If the lymphatic reflux caused enhancement of peritoneum, pleura or lung, we termed it as abnormal lymphatic perfusion.

The time from start of continuous nodal contrast-injection to initial enhancement of lymphatics in the venous junction were measured and the speed of contrast propagation was calculated.

Descriptive statistics were calculated and are given as median and range for continuous variables and count for discrete variables. Correlations between current clinical lymphatic manifestations and main imaging findings were assessed with Pearson’s correlation coefficient. The speed of contrast propagation was assessed using the Mann–Whitney test. Results were compared between both included major gene variants (*PTPN11* vs. *RIT1*).

### Ethical approval and informed consent

The presented study was approved by the institutional review board of the University of Bonn and hence all methods were performed in compliance with the ethical standards set in the 1964 Declaration of Helsinki as well as its later amendments. Written informed consent was waived.

## Results

### Patient cohort

Between January 2019 and April 2021 eleven patients with Noonan syndrome (5 female, 6 male; mean age 10.8 ± 16.4 years [range 2 months–49 years]) were referred for diagnostic work-up of lymphatic abnormalities. Seven patients were in their infancy; four were adults. The causative gene variant was a mutation of *PTPN11* (n = 5/11; 45.5%), *RIT1* (n = 5/11; 45.5%) and KRAS (n = 1/11; 9%). All but one patient had concomitant congenital heart disease.

All patients had a history of lymphatic abnormality from infancy, during which 3/11 patients (27%) presented with peripheral and genital edema (all with variants in *RIT1*). 10/11 (91%) had clinically proven chylothorax, 9/11 (82%) had signs of pulmonary lymphangiectasia, 2/11 (18%) had chylous ascites, while none of the patients had interstitial lymphangiectasia or lymphorrhea during infancy. In the four adults, peripheral edema had regressed in one during childhood, while clinical lymphatic manifestations were unchanged in the remaining patients. During adulthood the patient again developed peripheral/genital edema and one further patient showed late-onset peripheral/genital edema. Overall all adults presented with peripheral/genital edema. One patient developed chylocolporrhea and one protein-losing enteropathy as sign of intestinal lymphangiectasia. Detailed patient characteristics are given in Table 1.

**Table 1:** Patient characteristics.

Patient number	Sex	Age	Gene variant	Congenital heart disease	History of lymphatic abnormalities																				
					Infancy							Childhood							Adulthood						
					PE	GE	CT	PL	CA	IL	LR	PE	GE	CT	PL	CA	IL	LR	PE	GE	CT	PL	CA	IL	LR
1	M	19 years	*RIT1*	HCM	+	+	+	−	−	−	−	−	−	+	−	−	−	−	+	+	+	−	−	−	−
2	M	6 months	*PTPN11*	ASD, VSD, HAA	−	−	+	+	−	−	−														
3	M	21 years	*RIT1*	PS, ASD,	+	+	+	+	−	−	−	+	+	+	+	−	−	−	+	+	+	+	−	−	−
4	F	6 months	*PTPN11*	PS, ASD, VSD, HCM	−	−	+	+	−	−	−														
5	M	2 months	*PTPN11*	HCM	−	−	+	+	+	−	−														
6	F	2 months	*RIT1*	PS	−	−	+	+	−	−	−														
7	F	3 months	*RIT1*	HCM	−	−	+	+	−	−	−														
8	F	50 years	*PTPN11*	HCM	−	−	+	+	−	−	−	−	−	+	+	−	−	−	+	+	+	+	−	−	+
9	M	2 months	*KRAS*	HCM	−	−	−	+	−	−	−														
10	F	6 months	*PTPN11*	PS	−	−	+	+	+	−	−														
11	M	28 years	*RIT1*	None	+	+	+	−	−	−	−	+	+	+	−	−	−	−	+	+	+	−	−	+	−

F, female; M, male; ASD, atrial septal defect; CA, chylous ascites; CT, chylothorax; GE, genital edema; HAA, hypoplastic aortic arch; HCM, hypertrophic cardiomyopathy; LR, lymphorrhea; PE, peripheral edema; PS, pulmonary stenosis; PL, pulmonary lymphangiectasia; IL, intestinal lymphangiectasia; VSD, ventricular septal defect.

### T2-weighted imaging

**Figure 1:**
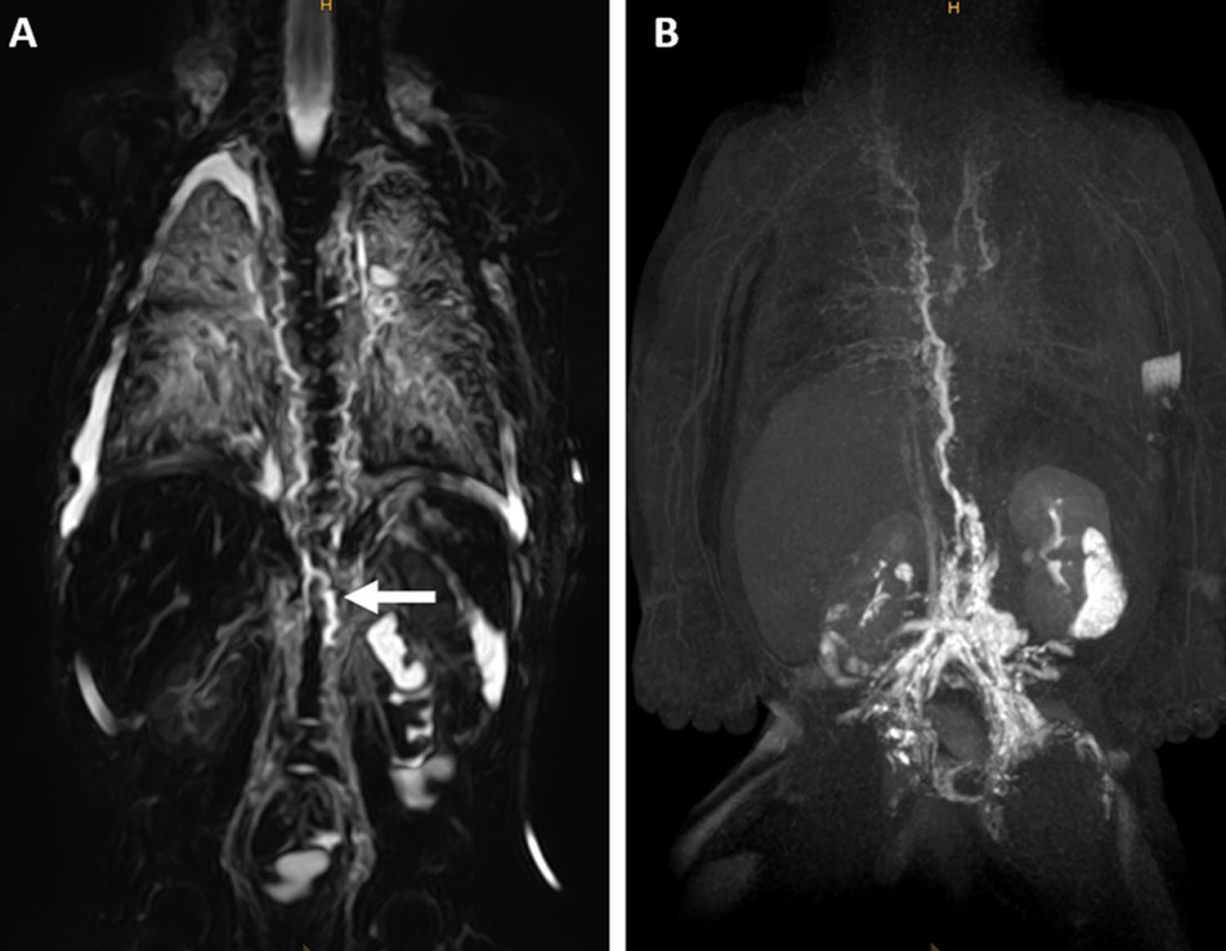
Two-month-old boy with Noonan syndrome-associated bilateral chylothorax and pulmonary lymphangiectasia. (**A**) Coronal, T2-weighted, non-contrast imaging demonstrates the right-sided course of the thoracic duct from the cisterna chyli (arrow) into the middle part of the thorax where it can no longer be distinguished from surrounding edema. Observe also the extensive pulmonary abnormalities and bilateral pleural effusions. (**B**) Coronal maximum-intensity projection (MIP) of a contrast-enhanced MR-lymphangiogram demonstrating dilated retroperitoneal lymphatics, the dilated cisterna chyli and thoracic duct in the lower and middle part of the thorax. The thoracic duct runs entirely on the right side and is only rudimentary in the upper part of the thorax. There is also cross-flow through mediastinal lymphatics to the left venous junction. There is only faint enhancement of cervical lymphatics without a discernable TD-venous junction of the thoracic duct. Observe also faint reflux into bilateral intercostal lymphatics and peribronchial lymphatics on the right.

T2w-imaging was available in all patients (Tables 2, 3). The cisterna chyli was identified in 10/11 cases (91%) (Fig. 1A). The TD, however, was visible continuously in only 1/11 cases (9%). It was completely absent in 1/11 (9%) and partially absent in 9/11 cases (82%). With 9/11 cases (82%) the lower part was the portion of the TD identified in most patients while the middle and upper thoracic as well as the cervical part was visible less frequently (Fig. 1A).

**Table 2:** Non-contrast-enhanced T2-weighted MRI imaging findings.

Patient number	Peripheral edema	Pleural effusions	Pericardial effusion	Ascites	Abdominal wall edema	Mesenteric edema	Periportal edema	Thoracic wall edema	Mediastinal edema	Pulmonary interstitial thickening	Axillary edema	Cervical edema
1	+	BL	+	+	+	−	−	−	−	−	−	BL
2	−	BL	−	−	−	−	−	−	+	BL	BL	BL
3	+	BL	−	−	−	+	+	BL	+	BL	−	R
4	−	BL	−	−	−	−	−	BL	+	BL	BL	BL
5	−	BL	−	+	−	+	−	−	+	BL	BL	BL
6	−	BL	−	−	−	−	−	BL	+	BL	−	BL
7	−	BL	−	−	−	−	−	BL	+	BL	−	BL
8	+	BL	−	−	+	−	−	−	−	L	−	−
9	−	−	−	−	−	−	−	−	+	BL	−	BL
10	−	L	−	−	−	−	−	BL	+	BL	BL	BL
11	+	BL	−	−	+	−	−	BL	+	−	−	−

BL, bilateral; L, left; R, right.

**Table 3:** T2-weighted imaging and dynamic-contrast enhanced MRL characteristics of central lymphatics.

Patient number	T2-weighted imaging							Dynamic contrast-enhanced MRL						
	CC	TD overall	TD lower	TD middle	TD upper	TD cervical	TD terminal	CC	TD overall	TD lower	TD middle	TD upper	TD cervical	TD terminal
1	+	Partially	+	−	−	+	L	+	Partially	+	−	−	−	−
2	+	Partially	+	+	+	−	−	+	Partially	+	+	+	−	−
3	+	Partially	+	+	−	−	−	+	Partially	+	+	+	+	−
4	+	Partially	+	+	+	+	−	+	Partially	+	+	+	+	−
5	+	Partially	+	+	−	−	−	+	Partially	+	+	−	−	−
6	Absent	Absent	−	−	−	−	−	Absent	Absent	−	−	−	−	−
7	+	Partially	+	−	−	+	L	+	Partially	+	−	−	+	BL
8	+	+	+	+	+	+	L	+	+	+	+	+	+	L
9	+	Partially	+	−	−	−	−	+	Partially	+	+	−	−	−
10	+	Partially	+	−	−	+	L	+	Partially	+	−	−	+	BL
11	+	Partially	−	−	+	+	L	+	Partially	+	−	−	−	−

BL, bilateral; CC, cisterna chili; L, left; R, right; TD, thoracic duct.

10/11 patients (91%) had pleural effusions, all but one were bilateral; additionally two patients had ascites and one had a pericardial effusion. Mediastinal edema and pulmonary interstitial thickening were also prevalent with 9/11 cases (82%) and correlated with the clinical presence of pulmonary lymphangiectasia. Cervical edema was seen in 9/11 cases (82%), extending into the axilla in four of these cases. Peripheral, abdominal wall, mesenteric and periportal edema was seen less often.

### DCMRL

**Figure 2:**
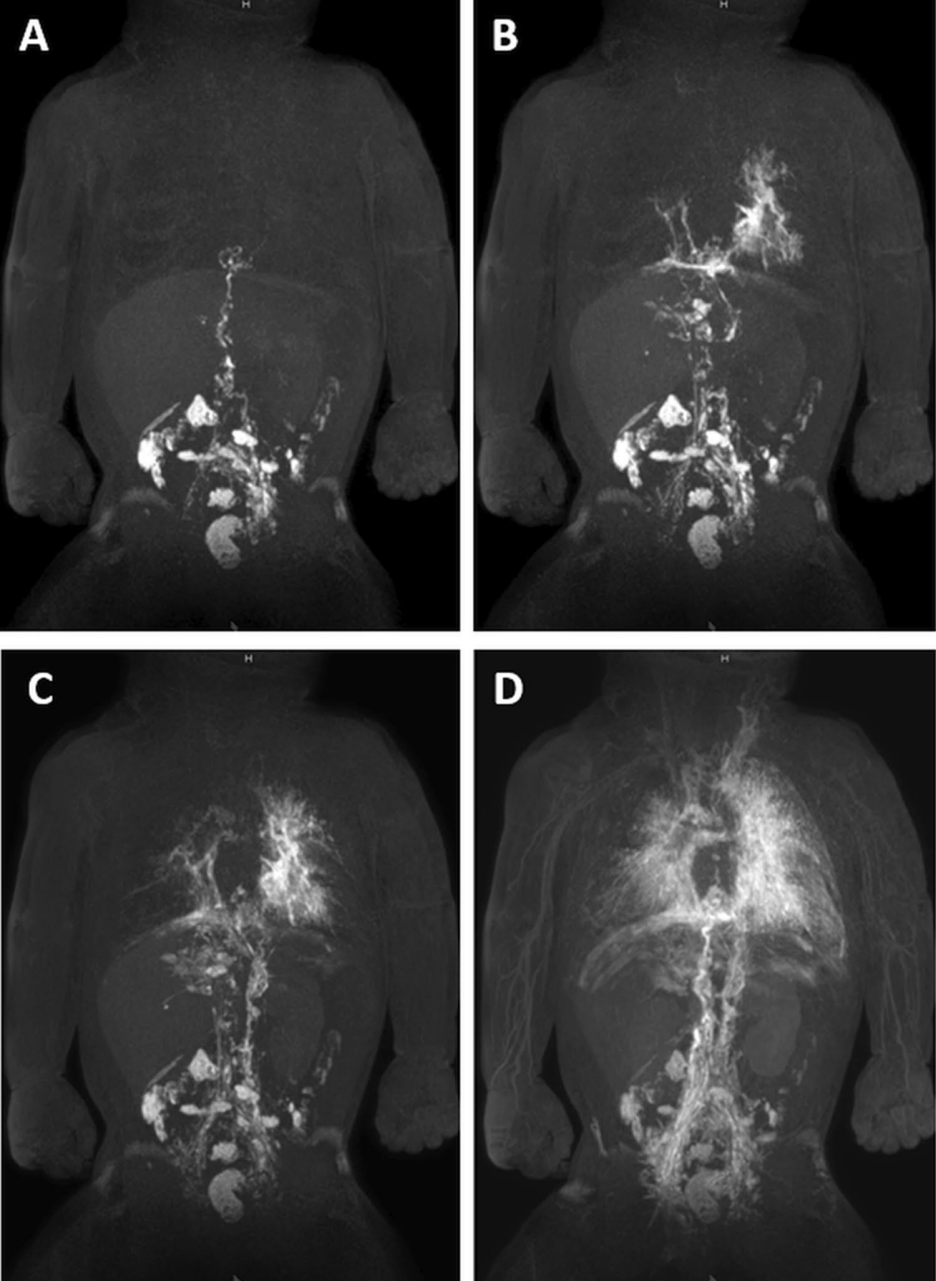
Three-month-old girl with severe pulmonary lymphangiectasia and bilateral Chylothorax. (**A**–**D**) Sequential dynamic maximum-intensity projection (MIP) images of coronal contrast-enhanced MR-lymphangiograms showing thoracic duct aplasia in the thorax with massive lymphatic reflux into both lungs. There is collateral flow through peribronchial, diaphragmatic and pleural / intercostal lymphatics and ultimately drainage towards the left venous junction with delayed enhancement of blood vessels.

**Figure 3:**
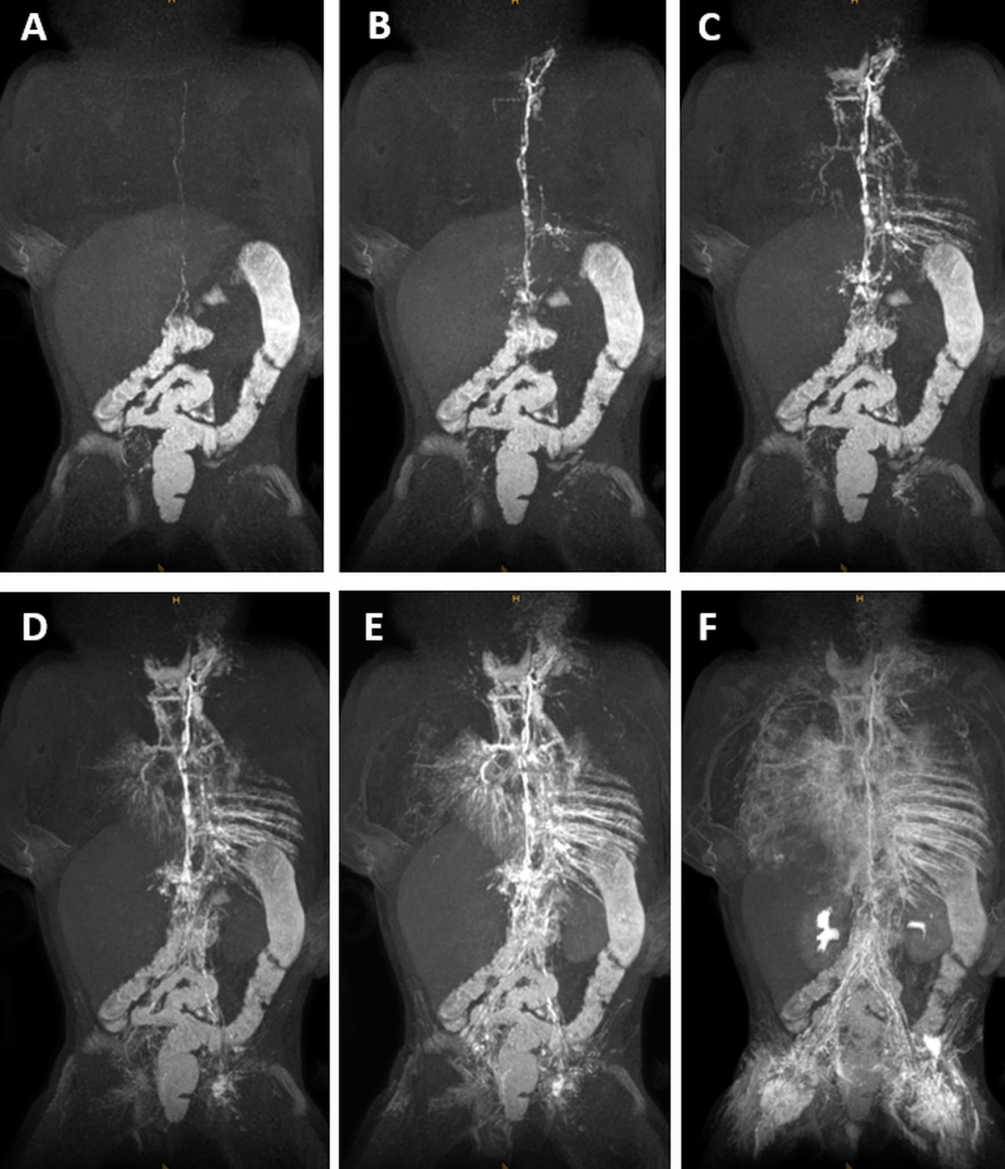
Six-month-old girl with bilateral chylothorax and pulmonary lymphangiectasia. (**A**–**F**) Sequential dynamic maximum-intensity projection (MIP) images of coronal contrast-enhanced MR-lymphangiograms demonstrating fast enhancement of retroperitoneal lymphatics and the thoracic duct. A TD-venous junction is not visible. Instead, the entire lymph flow from the thoracic duct drains into the left bronchiomediastinal trunk with massive reflux into mediastinal and peribronchial lymphatics. Observe also massive reflux into pleural / intercostal lymphatics (left more than right).

DCMRL was technically successful in all cases (Table 4). The cisterna chyli was identified in 10/11 patients (91%) (Fig. 1B). As on T2w-imaging, the TD was identified continuously in only 1/11 cases (9%); it was completely or partially absent in 1/11 (9%) and 9/11 cases (82%), respectively (Fig. 2). The lower part of the TD was identified in 10/11 cases (91%). Identification of the further parts of the TD decreased with direction of contrast-agent flow (Fig. 3). Although DCMRL identified the TD-venous junction less often than T2w-imaging (3 vs. 5), DCMRL revealed the venous junction to be bilateral in two cases which was not visible on T2w-imaging (Table 3).

**Table 4:** Dynamic contrast-enhanced MRL imaging findings.

Patient number	Leakage	Chylo-lymphatic reflux											Lymphatic perfusion		
		Peripheral	Genital	Abdominal wall	Mesentery	Mediastinum	Peribronchial	Intercostal	Internal mammary	Superficial thoracic	Cervical	Axilla	Peritoneum	Pleura	Lung
1	−	+	+	+	−	+	−	+	+	+	+	+	−	R	−
2	−	−	−	−	−	+	+	+	−	−	+	+	−	−	R
3	−	+	+	−	+	+	+	+	+	+	−	−	+	BL	BL
4	−	−	−	+	−	+	+	+	+	+	+	+	−	BL	BL
5	−	−	−	−	−	+	+	+	−	−	+	−	−	BL	BL
6	−	+	−	−	−	+	+	+	+	+	+	−	−	BL	BL
7	−	−	−	−	−	+	+	+	−	−	+	−	−	L	BL
8	Skin+vaginal	+	+	+	−	−	−	+	−	−	+	−	−	−	BL
9	−	−	−	+	+	+	+	+	+	−	+	+	−	BL	−
10	Thorax	−	−	−	−	+	+	+	+	+	+	+	−	R	BL
11	−	+	+	+	−	−	−	+	−	+	−	−	−	−	−

BL, bilateral; L, left; R, right.

**Figure 4:**
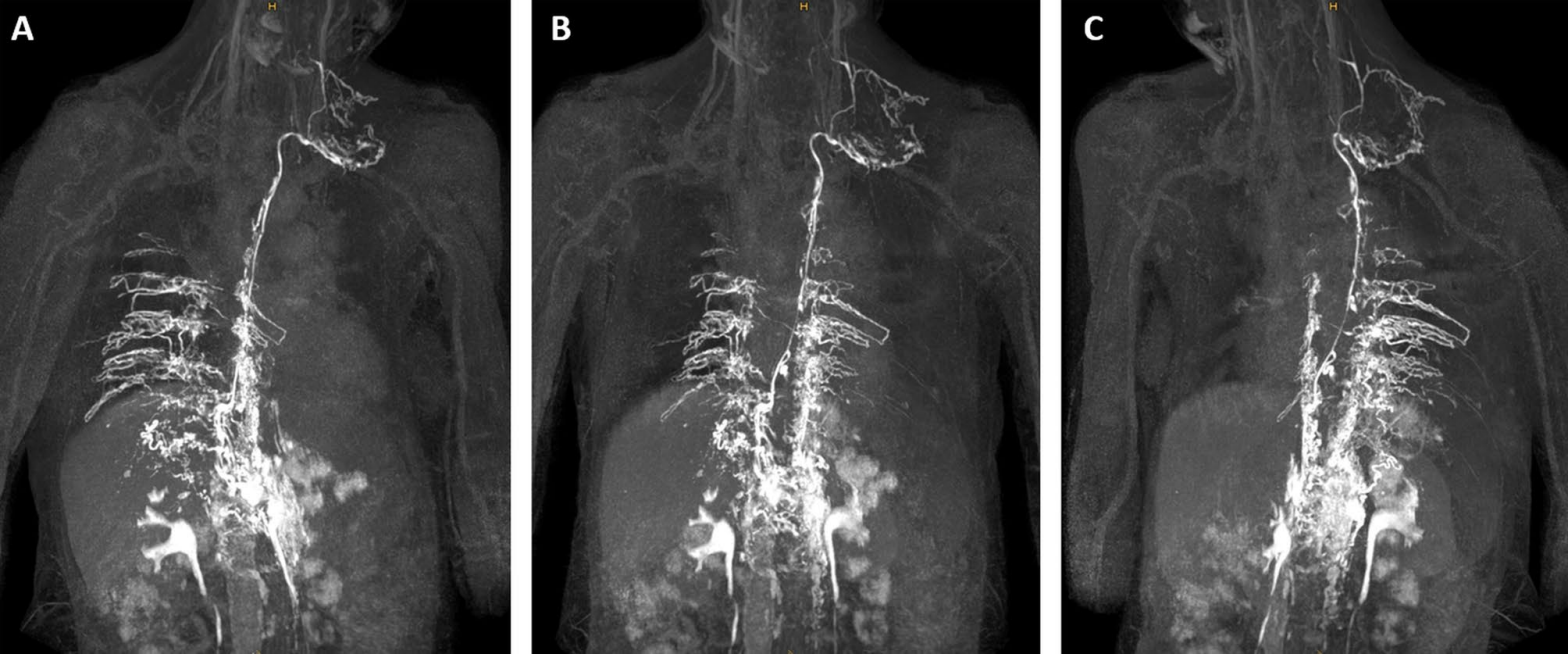
50-year-old woman with peripheral/genital edema and chylocolporrhea. (**A**) Right-anterior oblique, (**B**) anterior–posterior, (**C**) left-anterior oblique MIP reconstructions of contrast-enhanced MR-lymphangiograms demonstrating a thin, but continuous thoracic duct with termination in the left venous junction. There is bilateral lymphatic reflux into intercostal lymphatics.

**Figure 5:**
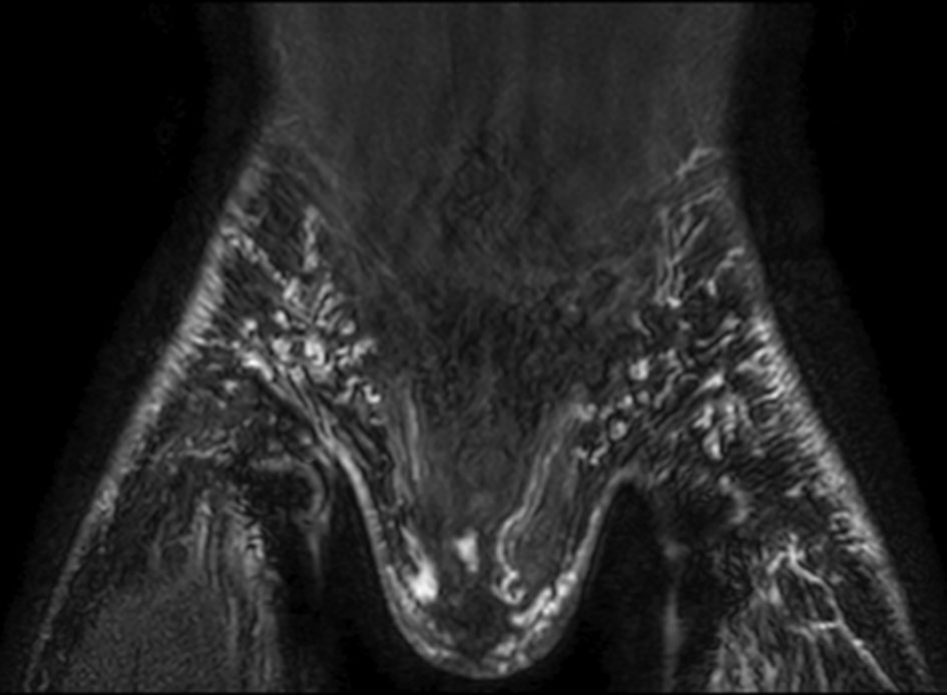
19-year-old male with peripheral/genital edema and chylothorax. Coronal contrast-enhanced MR-lymphangiogram of the inguinal region showing extensive lymphatic reflux from the inguinal nodal injection sites into superficial lymphatics of both legs, of the abdominal wall as well as into genital lymphatics.

**Figure 6:**
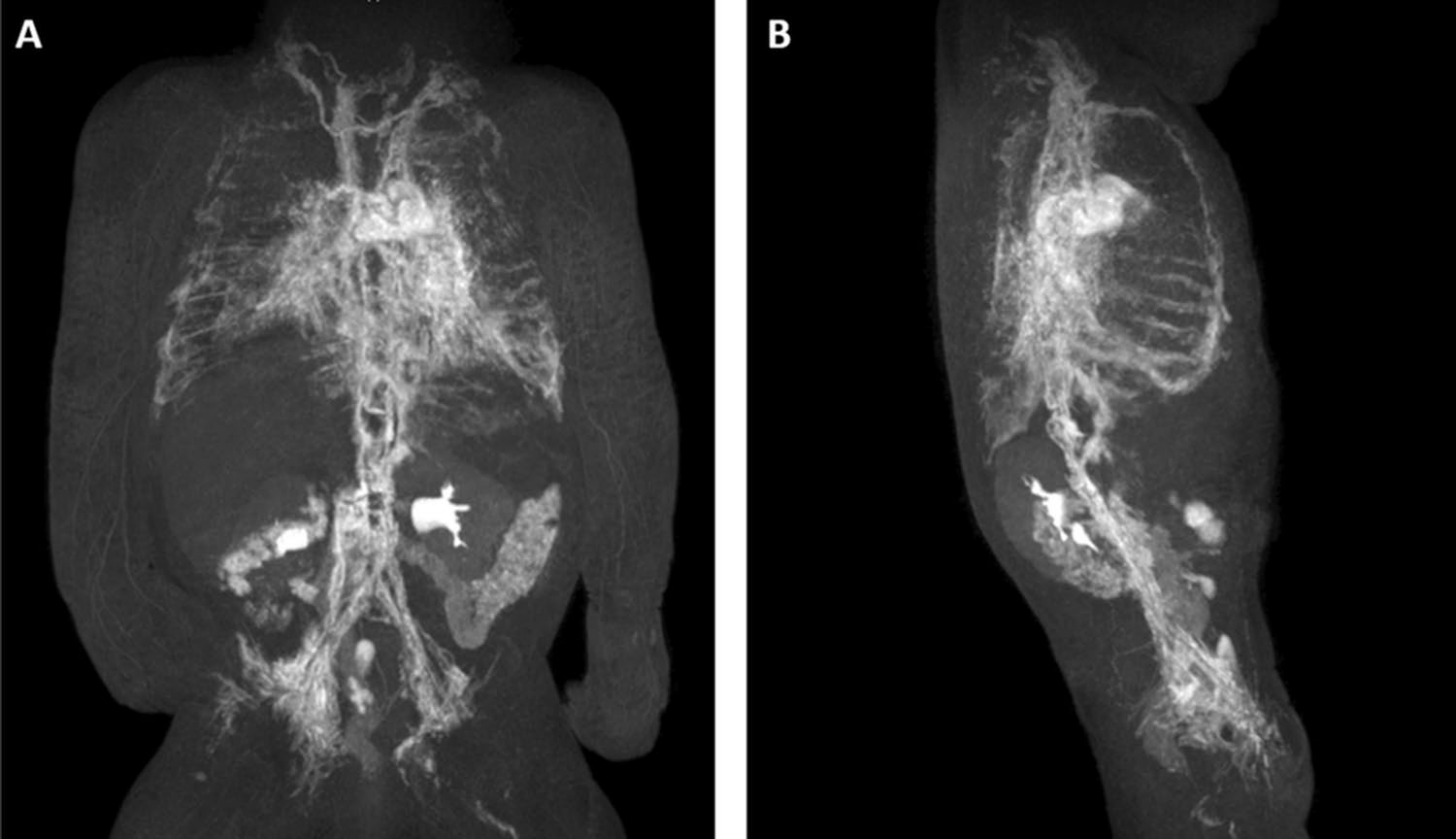
Two-month-old girl with chylothorax and pulmonary lymphangiectasia. (**A**) Anterior–posterior and (**B**) lateral MIP of contrast-enhanced lymphangiograms demonstrating absence of the thoracic duct in the middle and upper part of the thorax with lymphatic reflux into intercostal/pleural lymphatics, mediastinal as well as peribronchial lymphatics. Observe enhancement of the internal mammary trunks as well as lymphatics along the oesophagus.

Lymphatic reflux into intercostal lymphatics was observed in all patients (Fig. 4). Reflux into mediastinal and peribronchial lymphatics was also seen in the majority of cases (9/11 (82%) and 8/11 (73%), respectively) (Figs. 1, 2, 3). Cervical lymphatic reflux was seen in 9/11 patients (82%). Retrograde flow into peripheral and genital lymphatics was visible in 5/11 (45.5%) and 4/11 cases (36%), respectively (Fig. 5). Lymphatic collaterals in the abdominal or thoracic wall were seen in 5/11 (45.5%) and 6/11 patients (54.5%), respectively. Diaphragmatic lymph vessels and the internal mammary truncs showed enhancement in 5/11 cases (45.5%) (Fig. 6). Finally, we observed lymphatic flow from the abdomen into the thorax via paraoesophageal lymphatics in 3/11 patients (27%) (Patient No. 6, 7 and 9) (Fig. 6).

Abnormal lymphatic perfusion of lung parenchyma was seen in 8/11 patients (73%) (7 bilateral, 1 unilateral) and of the pleura in 8/11 patients (73%) (5 bilateral, 3 unilateral). Peritoneal lymphatic perfusion was only seen in one patient who did not have ascites at the time.

Overall active contrast-medium extravasation was observed in 2/11 patients (18%). In one female adult vaginal lymphatic leakage was identified due to reflux from the iliac lymphatics. In one infant diffuse active extravasation into the left pleural effusion was seen.

Complications of nodal contrast-application were not observed.

**Figure 7:**
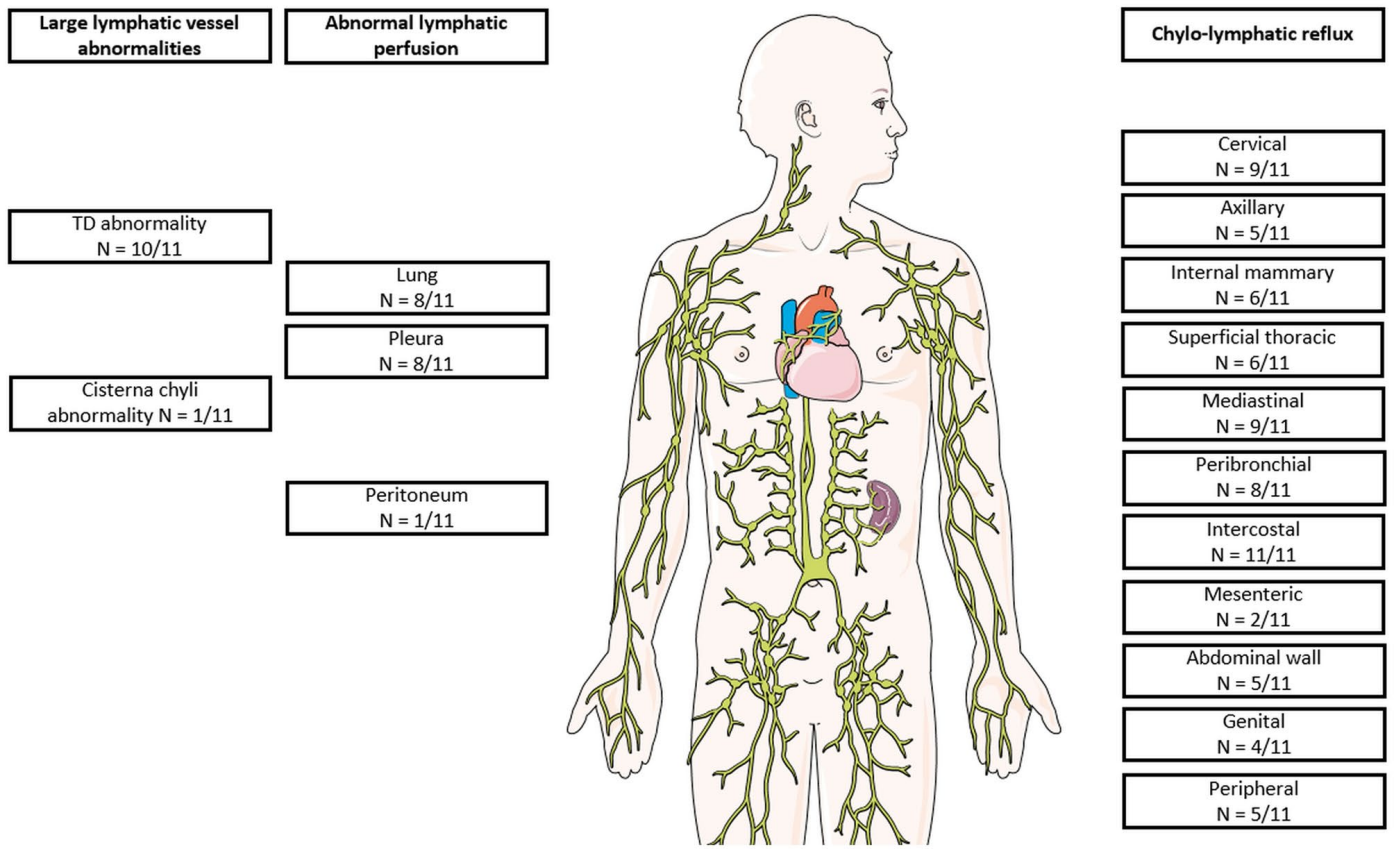
Overview of major imaging findings on DCMRL (image: smart.servier.com).

Figure 7 gives an overview of main imaging findings.

### Relationship between imaging findings and current symptoms

Peripheral reflux showed a strong correlation with peripheral edema as it was observed in all 4 patients with and only in one patient without peripheral edema (r = 0.828, p = 0.002). Genital reflux was only observed in patients presenting with genital edema (r = 1.000, p < 0.001). Abnormal pleural lymphatic perfusion was seen in 7/10 in patients with chylothorax, but also in the patient without chylothorax. Than again it was missing in the remaining 3/10 patients with chylothorax (r = − 0.194, p = 0.568). Abnormal pulmonary lymphatic perfusion was strongly correlated with pulmonary lymphangiectasia (8/9 patients; r = 0.770, p = 0.006).

In the patient with local vaginal lymphorrhea, lymphatic reflux into the inner genital was see on DCMRL.

**Figure 8:**
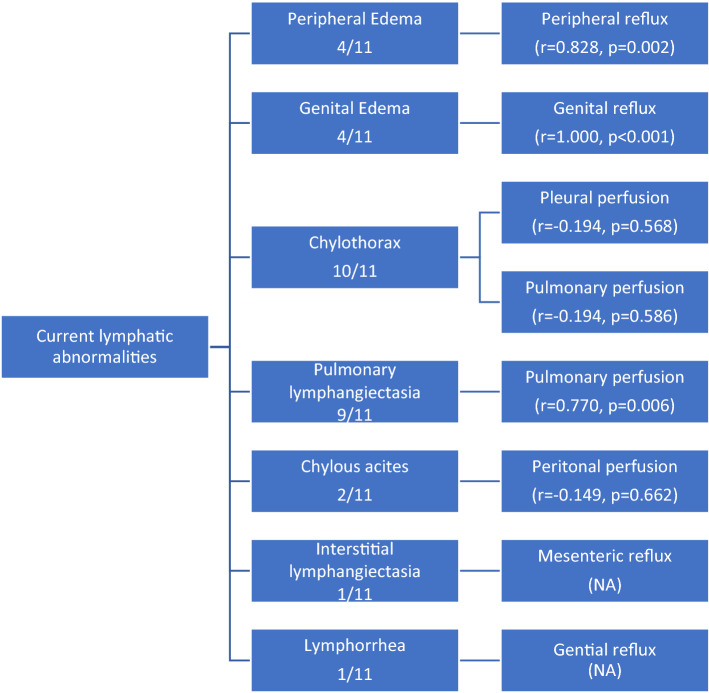
Correlation of current symptoms and major imaging findings (statistical results of Pearson’s correlation).

Figure 8 gives an overview of possible relationships between imaging findings and current symptoms.

### Speed of contrast propagation

Enhancement of the uppermost visible lymphatics was observed within 1 min to > 30 min. Concerning the speed of contrast propagation, two different groups were identified: In 6/11 patients (54.5%) the speed was above 10 cm/min (median 19.7 cm/min, range 10.5–23 cm/min), while it was considerably below 10 cm/min in 5/11 patients (45.5%) (median 2.3 cm/min, range 1.9–3.6 cm/min) (p = 0.026). In the four adults speed of contrast propagation was below 10 cm/min in 3/4.

### Comparison of PTPN11 and RIT1

Peripheral and genital edema was more prevalent in patients with *RIT1*-mutations than in patients with *PTPN11*-mutations during infancy (*PTPN11*: n = 0/5, *RIT1*: n = 3/5) and childhood (*PTPN11*: n = 0/1, *RIT1*: n = 2/3). Both cases of chylous ascites during infancy were observed in patients with *PTPN11*-mutations. All other clinical parameters did not show considerable differences between both groups.

On T2w-imaging thoracic wall edema was seen in 4/5 patients with *RIT1* and only in 2/5 with *PTPN11*. In contrast, axillary edema was present in 4/5 patients with *PTPN11*, but in none of the patients with *RIT1*.

When comparing the speed of contrast propagation, 4/5 patients with *PTPN11*-mutations showed values above 10 cm/min (median 20.2 cm/min, range 19–23 cm/min). In contrast, 4/5 patients with *RIT1*-mutations had values below 10 cm/min (median 2.3 cm/min, range 1.9–3.6 cm/min).

## Discussion

We evaluated the central lymphatic system (CLS) in patients with Noonan syndrome who had a history of lymphatic abnormalities employing MR lymphangiography. The main results of the study are: First, DCMRL demonstrated extensive abnormalities of the CLS, even if patients only had localized clinical signs of lymphatic dysfunction. However, lymphatic abnormalities were most pronounced in the thorax. Second, lymphatic reflux into intercostal lymph vessels was the most prevalent single imaging sign. Further frequent findings of the disorder were abnormal pulmonary/pleural lymphatic perfusion and TD-dysgenesis or agenesis. Third, considerable imaging abnormalities of the CLS were also present in patients with *RIT1*-mutations. Although clinical signs of lymphatic abnormalities in patients with *RIT1*-mutation are known, corresponding imaging results have not been reported before. Fourth, clinical lymphatic manifestations differed slightly between patients with *PTPN11*- and *RIT1*-mutations with peripheral edema being more prevalent in patients with *RIT1*-mutations.

Noonan syndrome is associated with different lymphatic abnormalities^2,9–17^. We describe a cohort of pediatric and adult Noonan-patients with a wide spectrum of clinical lymphatic pathologies in whom DCMRL demonstrated widespread morphological and functional CLS abnormalities. All had a history of chylothroax and/or pulmonary lymphangiectasia in common. Accordingly, anomalies of thoracic lymphatics were the most severe on DCMRL with retrograde flow in thoracic lymphatics as well as lymphatic perfusion of pleura and/or lung. This is in line with previous reports in patients with chylothorax and/or pulmonary lymphangiectasia^14,17,20^. Interestingly, while we observed a strong correlation between abnormal pulmonary lymphatic perfusion and the presence of pulmonary lymphangiectasia, the same was not true for pleural or pulmonary lymphatic perfusion and the presence of a chylothorax. Abnormal pleural perfusion was also observed in the patient without chylothorax. Furthermore, three patients currently presenting with a chylothorax did not show abnormal pleural lymphatic perfusion. These findings as well as the observation that the side of abnormal pleural perfusion did only partially fit the side of chylothorax points to a more complex pathophysiology of chylothorax development in these patients.

Reflux can also occur into other parts of the body (e.g. towards the genital in chylocolporrhea^31^ or towards the kidneys in chyluria^24^). Reflux into peripheral lymphatics was mainly seen in patients with peripheral edema in our cohort. In this respect, also hypo- or hyperplastic peripheral lymphatics have previously been described^19,32^. Genital lymphatic reflux also correlated strongly with the presence of genital lymphedema and vaginal lymphorrhea in our cohort. The specific lymphatic flow pattern therefore varies and seems to correlate with the individual clinical presentation.

Abnormalities of the TD itself are a more consistent imaging finding present in most Noonan-patients with lymphatic abnormalities^13,14,22,23,25,32^ and were also observed in all but one patient in our cohort. As TD-dysgenesis or agenesis considerably impairs central lymphatic run-off, other observations like lymphatic reflux may primarily be consequences of collateralization of TD-abnormalities. Usually the TD drains the lymph from the lower (infradiaphragmatic) half of the body, the posterior peritoneum as well as the posterior parietal and posterior diaphragmatic pleura. The internal mammary trunks drain the lymph from the anterior parietal and anterior diaphragmatic pleura, peritoneum, the anterior abdominal wall as well as parts of the liver^33^. The lung drains its lymph via pleural lymphatics into perivenous lymphatics as well as via peribronchial lymphatics towards the hilum^34^. From the hilum lymphatic flow follows the bronchomediastinal trunks towards the venous junctions where it drains in close proximity to or together with the TD and the internal mammary trunks^33,35^. There are therefore several anatomically preformed interconnections between thoracic lymph vessels that may serve as collateral pathways in TD-dysgenesis—provided that lymphatic valves are incompetent or missing^13^. Therefore, lymph from the lower body may drain retrogradely through pulmonary lymphatics, lymphatics of the parietal pleura (which are organized along the intercostal spaces^36^ and diaphragm) into the internal mammary trunks and bronchomediastinal trunks to drain into the venous junctions. These anatomical conditions can explain flow patterns and consequently clinical symptoms.

So far no clear genotype–phenotype correlations have been identified for different mutations in Noonan syndrome. Data from one report suggests that patients with *RIT1*-mutations may have a higher incidence of lymphatic dysfunction in the fetal period (nuchal edema, chylothorax, hydrops) and later in life (late onset peripheral/genital lymphedema, chylothorax, intestinal lymphangiectasia)^2^. This is in line with the fact that *RIT1* is overrepresented in our cohort of Noonan patients with lymphatic abnormalities (45%), while it only accounts for 6.8% of all cases with Noonan syndrome. In this respect, we also observed differences in clinical presentation and flow patterns on DCMRL between patients with different gene-variants. Patients with *PTPN11*-mutations showed a slightly higher rate of pulmonary lymphangiectasia during infancy than patients with *RIT1*-mutations who showed a higher prevalence of peripheral/genital edema. These differences, different thoracic wall edema distributions and different flow dynamics (preferentially fast flow in *PTPN11* vs. slow flow in *RIT1*) may point to a different pathophysiological background in both patients groups.

Apart from the genetic background, patient age may also play a role in clinical presentation^2^, which was dominated by peripheral/genital edema and peripheral leakage in adults in our cohort although these patients also had a history of chylothorax and/or pulmonary lymphangiectasia since infancy. This may indicate changes in lymphatic flow and the capacity of lymphatic collateral networks over the course of years. However, due to the small and heterogeneous patient cohort this conclusion cannot be drawn with certainty. Further research into dynamic changes of lymph flow seems to be warranted.

Different imaging techniques—traditionally lymphoscintigraphy or X-ray lymphangiography—may yield information on different aspects of the same disease spectrum. Our experience with T2w- and DCMRL demonstrate that to a certain degree both imaging techniques yield comparable results, especially concerning the identification of TD-dys-/agenesis. However, although T2w-imaging is best suited to identify effusions and the distribution of edema, pathological lymphatic flow patterns can only be evaluated by contrast-enhanced imaging. We therefore advocate to employ T2w- as well as DCMRL for a comprehensive evaluation of the CLS, especially when therapeutic decisions are to be based on the imaging results.

In future, detailed imaging evaluation of lymphatic flow abnormalities may help guide treatment approaches. Interventional treatment of lymphatic abnormalities have been increasingly employed in recent years^37–40^ and may also be suitable in carefully selected patients with Noonan syndrome as recently reported in chylocolporrhea^31^. However, especially as demonstrated by poor/fatal outcomes after embolization in several patients reported by Biko et al.^17^, special care must be taken to select suitable candidates. DCMRL may therefore be a valuable clinical tool for treatment planning in these patients. As TD-abnormalities are a common finding, microsurgical lymphatico-venous anastomoses may also be advantageous in selected patients^41^. Finally, initial experiences on a single patient level exists for medical treatment targeted at defects in the RAS-pathway. In one case of an infant with Noonan syndrome and protein-losing enteropathy, treatment with a MEK-inhibitor led to resolution of symptoms and remodeling of the CLS^42^. Similar beneficial results had previously been published for Noonan-associated hypertrophic cardiomyopathy^43^.

The present study has several limitations. First, data were analyzed retrospectively in a small patient cohort owing to the rarity of clinically manifest lymphatic abnormalities in Noonan-patients. We therefore refrained from in-depth statistical analysis, especially in small subgroups. Second, the patient cohort was rather heterogeneous concerning causative genes, clinical manifestations and age. However, all patients had genetically proven Noonan syndrome including patients with a mutation in one of the major genes (*RIT1*) in which so far no imaging results have been reported. Third, only patients with symptomatic lymphatic abnormalities were included introducing a selection bias. Further multi-institution studies are needed to evaluate the incidence of—also asymptomatic—lymphatic abnormalities in patients with Noonan syndrome. Fourth, the measurement of the speed of contrast propagation is limited by the fact that injections were done by hand. Although we tried to standardize injection speed as far as possible, this may to a certain degree have influenced measurement results. Furthermore, “normal” speed of contrast propagation with the employed examination technique are not established. However, there was a marked difference in contrast propagation between patients with *PTPN11* and *RIT1* mutations, which is in interesting observation warranting further investigation.

In conclusion, children and adults with Noonan syndrome presenting with chylothorax and/or pulmonary lymphangiectasia have widespread central lymphatic abnormalities. Intercostal lymphatic reflux, thoracic duct abnormalities and pulmonary/pleural lymphatic perfusion are typical abnormalities. There are possible phenotypical differences of lymphatic manifestations between patients with *PTPN11* and *RIT1*-mutations. MR-lymphangiography can elucidate individual flow patterns and may therefore in future be helpful for targeted treatment planning. Further research in larger multicenter studies is warranted to further our understanding of this complex disorder.
